# Gynecologic cancer survivor preferences for long-term surveillance

**DOI:** 10.1186/s12885-018-4313-x

**Published:** 2018-04-03

**Authors:** Matthew Schlumbrecht, Charlotte Sun, Marilyn Huang, Andrea Milbourne, Diane Bodurka

**Affiliations:** 1 Division of Gynecologic Oncology, The University of Miami Sylvester Comprehensive Cancer Center, 1121 NW 14th St, Suite 345, Miami, FL 33136 USA; 2 Department of Gynecologic Oncology and Reproductive Science, The University of Texas MD Anderson Cancer Center, Unit 1362, 1515 Hermann Pressler Dr, Houston, TX 77030 USA

**Keywords:** Gynecologic cancer, Preferences, Surveillance, Ovarian cancer, Endometrial cancer

## Abstract

**Background:**

With ongoing healthcare reform and shrinking numbers of oncologists, appropriate triaging of gynecologic cancer survivor care is crucial. Input from patients is a necessary part of this task. The objective of this study was to assess the preferences of gynecologic cancer survivors for surveillance after the completion of treatment.

**Methods:**

A 38-item questionnaire was developed and launched in conjunction with the Foundation for Women’s Cancer (FWC). All women who registered as gynecologic cancer survivors with the FWC were invited to participate. Patients were asked about physician preferences for multiple symptoms and diagnoses, and when they felt comfortable transferring care out of their oncologists’ offices. Analyses were performed with chi-square and logistic regression.

**Results:**

Six hundred twenty four patients completed the questionnaire. Sixty six percent had ovarian cancer, and 86% were primarily treated by a gynecologic oncologist. Fifty seven percent of the respondents reported being unwilling to see a physician other than their oncologist for survivorship care at any time. Women age > 60 years were less willing to leave their oncologists for survivorship care at any time compared to younger women (OR 1.53 [95% CI 1.03–2.27], *p* = 0.03). Ovarian cancer survivors were also more likely to report a desire to stay with their oncologists compared with uterine cancer survivors (*p* < 0.001). With few exceptions, respondents preferred management of non-oncologic medical problems by their oncologists.

**Conclusions:**

Gynecologic cancer survivors prefer that their oncologists remain heavily involved in survivorship care. Reconciling patient needs with physician and financial constraints will be a challenge as the survivor population continues to grow.

## Background

In 2016, it was estimated that there were over 1.2 million survivors of gynecologic malignancy in the United States [1]. With improvements in the treatment of these diseases, the number of cancer survivors will continue to grow. Meeting the challenges of this population, including the physical and psychosocial sequelae of cancer treatment, is well recognized. In fact, the Institute of Medicine (IOM) released guidelines for cancer survivorship more than ten years ago [2]. Four areas which have been advocated as part of survivorship care include surveillance for the most recent cancer, screening for cancers other than the primary malignancy, general preventative health, and the management of medical comorbidities both resulting from, and independent of, previous cancer treatment [3]. Because several of these areas are not oncology-specific, there has been growing interest in the role of primary care practitioners (PCPs) and benign obstetrician/gynecologists (OB/GYN) in the care of gynecologic cancer survivors.

Studies have demonstrated that cancer survivors, PCPs, and oncologists all expect to be heavily involved in the survivorship phase of the cancer continuum after definitive treatment is completed [3]. However, a fundamental drawback of this research is that few gynecologic oncology patients and OB/GYNs are included, so the expectations for these groups are relatively unknown. Studies about gynecologic cancer survivor preferences for follow-up are actually few when compared to other disease sites. However, several consistent themes have arisen from such research. In 2009, Kew et al. [4] surveyed a small cohort of survivors about preferences for care. Eighty-nine percent (89%) preferred to see a hospital doctor in lieu of either a specialist nurse or general practitioner. The majority of women also thought that the examination was the most important part of the visit. A subsequent meta-analysis demonstrated that women prefer having follow-up done by a specialist, but the type of specialist is not explicitly described [5]. Greimel et al. [6] also reported that the majority of women thought the most important component of the visit was the gynecologic examination.

Gynecologic oncology survivors are a special population of women who experience treatment side effects and quality of life issues that set them apart from other cancer survivors, including sexual dysfunction and radiation-induced pelvic fractures, and their care thus requires expertise in female pelvic medicine [7]. At present, the Society of Gynecologic Oncology recommends taking a thorough history, performing pelvic examinations (to include both vaginal and rectal exams), and educating cancer survivors as the most effective ways to detect cancer recurrence; routine imaging is not advocated [8]. As the majority of non-gynecologic practitioners do not routinely perform pelvic examinations, many gynecologic cancer patients will undergo survivorship care with those who do. Given the pressing need to establish survivorship programs for this unique and growing population, assessing survivor preferences for follow-up, particularly in regards to the role of benign OB/GYNs, is critical to effectively triage patients to appropriate non-oncologic physicians, and to understand patient expectations for follow-up.

The primary objective of this study was to complete an exploratory investigation of gynecologic cancer survivor preferences for long-term survivorship care, and to specifically evaluate the type of physician survivors prefer to direct such care. The secondary objectives include an assessment of care preferences for medical problems commonly experienced by survivors, though not necessarily related to their cancer diagnoses; and to determine sociodemographic factors which may affect preferences for care. We hypothesized that gynecologic cancer survivors will prefer their oncologists as the primary clinicians for cancer survivorship needs.

## Methods

After approval from the University of Texas M.D. Anderson Cancer Center Institutional Review Board, a 38-item questionnaire was developed de novo to assess patient preferences for survivorship care. The questionnaire (Appendix A) consisted of both single answer and Likert-style questions tailored specifically to a population of gynecologic oncology patients, and was based on themes emphasized by previous authors investigating preferences for follow-up in medical oncology patients [3]. Questions included data about patient demographics (age, region of residence, insurance coverage, and distance to treating physicians), clinical history (type of cancer, date of diagnosis, and time since conclusion of treatment), recurrence, and preferences for the care of 23 different medical, surgical, and treatment-related conditions. Specifically, the questionnaire inquired about who the patient preferred to manage each specific condition – an OB/GYN or an oncologist. Patients were also asked who they preferred to provide their overall survivor care, including cancer surveillance and treatment of general health conditions, regardless of financial limitation: oncologist, internist, family practitioner, or OB/GYN. Advanced practice providers were not included as an option given variability in practice by region, and to remain consistent with previously reported results [9]. Participants were also asked how many years after completing cancer treatment would they be willing to see an OB/GYN instead of an oncologist for follow-up. A field for free text responses was provided for patients to explain their preferences for follow-up if they desired. The questionnaire was designed to be intelligible on an 8th grade reading level.

A small group of inpatients at our institution (*n* = 10) served as a pilot cohort for the questionnaire to assess understandability of the questions. Following minor grammatical modifications after receiving feedback from the pilot group, the questionnaire was finalized and converted to an electronic format utilizing SurveyMonkey©. A link to the questionnaire was then posted on the website for the Foundation for Women’s Cancer (FWC). The Foundation for Women’s Cancer is a philanthropic group which supports research, education, and public awareness of women’s cancers. As part of its mission, the foundation conducts gynecologic survivorship courses at a number of sites annually. Participants in the FWC survivorship courses were made aware of the study and encouraged to go to the website (http://www.foundationforwomenscancer.org/) to participate. Women were eligible if they had a personal history of a gynecologic malignancy, could read and understand English, had completed treatment, and had not been diagnosed with recurrent disease. Questionnaires were anonymous, and no incentives were provided for participation in the study. Consent for participation was implied if patients elected to complete the survey, and such implied consent was deemed acceptable by The University of Texas MD Anderson Cancer Center Institutional Review Board. The link to the questionnaire remained open for twelve weeks, after which time no additional data were collected for inclusion in the overall analysis. Data were then compiled into a central database.

Statistical analyses were performed using STATA IC 13.0 (StataCorp, College Station, TX) and IBM SPSS Statistics Version 23. Descriptive statistics were used to characterize the data (frequencies, percentages). For a host of medical and cancer-related conditions, logistic regression was used to evaluate the influence of key independent variables (age, cancer type, etc.) on respondents’ preferences for a gynecologist vs. oncologist (including gynecologic oncologist). Our independent variables were categorical (age groups, treatment physicians, cancer type, insurance type, distance), and we defined our dependent variable as “preferred physician” (i.e. oncologist versus OB/GYN). Chi-square analyses (or Fisher’s exact test, when appropriate) were also used to determine associations between categorical variables. All tests were two-sided, and *p*-values < 0.05 were considered statistically significant.

## Results

Six hundred and twenty-four (624) patients completed the online questionnaire over the twelve weeks data were collected. Of these, 44 questionnaires were mostly incomplete and discarded. One hundred thirty-one (131) respondents had developed recurrent disease, thus making them ineligible. A total of 449 (72.0%) questionnaires were therefore available for analysis, and from these all data points were used, even if the overall questionnaire was not complete. Demographic characteristics are shown in Table 1. The largest proportion of women was diagnosed at greater than 50 years of age, although there was representation from a large variety of age groups. In fact, five patients diagnosed at ages 19 and younger participated. The majority of women had ovarian, fallopian tube, or primary peritoneal cancer (65.9%), while 114 patients (25.4%) had endometrial/uterine cancer. A gynecologic oncologist was the primary treating physician in more than 85% of cases. For more than half of the respondents, their oncologists’ offices were relatively close to their homes (< 20 miles) and were considered easily accessible. Thirty-six percent (35.9%) of the women did not have an established OB/GYN.

**Table 1:** Respondent demographics (*N* = 449)

Age at Diagnosis	< 40 years	40 (8.9%)
	40–49 years	105 (23.4%)
	50–59 years	187 (41.6%)
	60–69 years	92 (20.5%)
	≥70 years	25 (5.6%)
Primary Site of Disease	Vulva/vagina	8 (1.8%)
	Cervix	30 (6.7%)
	Endometrial/uterine/GTN	115 (25.5%)
	Ovary/fallopian tube/peritoneal	296 (66.0%)
Area of Residence	Northeast/Mid-Atlantic	100 (22.2%)
	South Atlantic	82 (18.3%)
	South Central	45 (10.0%)
	North Central	130 (29.0%)
	Mountain/Pacific	82 (18.3%)
	International	9 (2.2%)
Insurance Coverage	Medicaid/Medicare	98 (22.2%)
	PPO/HMO	319 (72.2%)
	Uninsured	8 (1.8%)
	Military/Other	17 (3.8%)
Physicians involved in care	Gynecologic Oncologist	429 (95.5%)
	Medical Oncologist	139 (31.0%)
	Radiation Oncologist	94 (20.9%)
	Other/unsure	86 (19.2%)
Distance to oncologist’s office	≤20 miles	240 (54.7%)
	21–50 miles	127 (28.4%)
	51–100 miles	41 (9.3%)
	> 100 miles	31 (7.1%)
How convenient to go to oncologist’s office?	Very convenient	166 (37.6%)
	Slightly convenient	43 (9.7%)
	Neither convenient nor inconvenient	38 (8.6%)
	Slightly inconvenient	108 (24.4%)
	Very inconvenient	87 (19.7%)
Which office is closer to your residence?	Oncologist	37 (8.4%)
	OB/GYN	109 (24.8%)
	Oncologist and OB/GYN equidistant	132 (30.1%)
	Don’t have OB/GYN	161 (36.7%)

Patient preferences for survivorship care by specific medical issue are presented in Table 2. For general medical conditions, such as hypertension, high cholesterol, diabetes, weight loss, and smoking cessation, more than two-thirds of the patients reported no preference for which physician managed the problem. Most patients preferred that their oncologist treat likely oncology-related issues, including surveillance for cancer recurrence, lymphedema, bowel obstruction, and fistula. Interestingly, 17% of women preferred their oncologists to manage their fertility issues (versus 44% OB/GYN, *p* < 0.01), while 29% preferred their oncologists to manage issues of sexual dysfunction (versus 46.4% OB/GYN, *p* < 0.01) and 29% management of menopause symptoms (versus 47.1% OB/GYN, *p* < 0.01). Overall, 75 % (75%) of patients wanted their oncologists to manage abnormal pap smears, versus a 19% preference for an OB/GYN (*p* < 0.01), although if a patient received care from a medical oncologist, she was much less likely to want the medical oncologist to manage abnormal pap smears (OR 0.52 [95% CI 0.31–0.88], *p* = 0.01) . In general, for those women who did have a preference for management of specific medical problems, they were more likely to favor having an oncologist manage their non-oncologic medical problems if they had previously received care from a medical oncologist, and specifically for diabetes (OR 3.55 [95% CI 1.59–7.96], *p* = 0.002), hypercholesterolemia (OR 4.13 [95% CI 1.96–8.70], *p* < 0.001), smoking cessation (OR 2.94 [95% CI 1.18–7.35], *p* = 0.02), hypertension (OR 3.12 [95% CI 1.54–6.31], *p* = 0.002), and weight loss counseling (OR 2.84 [95% CI 1.41–5.74], *p* = .0004). Age, insurance type, and distance to the care provider’s office were not associated with preferences for follow-up care for any of the medical problems queried.

**Table 2:** Survivor preferences for care by medical issue. Totals vary for each condition due to variable participant responses to questions

	Oncologist (includes gynecologic oncologist)	Obstetrician/gynecologist	No preference/No answer
	Number (%)	Number (%)	Number (%)
Diabetes	63 (16)	57 (14)	280 (70)
Thyroid Problems	97 (24)	61 (15)	244 (61)
Quitting smoking	43 (11)	45 (12)	300 (77)
Weight loss	76 (19)	82 (20)	251 (61)
High cholesterol	65 (16)	77 (20)	254 (64)
Depression/Anxiety	108 (27)	75 (19)	221 (54)
High blood pressure	75 (19)	77 (19)	250 (62)
Anemia	136 (35)	62 (15)	196 (50)
Kidney problems	144 (36)	64 (16)	190 (48)
Problems urinating	154 (38)	110 (27)	138 (35)
Problems with bowel movements	185 (45)	90 (22)	134 (33)
Passing stool from your vagina	212 (53)	131 (32)	61 (16)
Passing urine from your vagina	203 (50)	134 (33)	68 (17)
Bleeding from your rectum	238 (59)	77 (19)	88 (22)
Blood in your urine	220 (55)	92 (23)	88 (22)
Surveillance for cancer recurrence	356 (85)	43 (12)	18 (3)
Low bone density	124 (31)	116 (28)	165 (41)
Excessive swelling in your legs (lymphedema)	220 (54)	58 (14)	127 (31)
Menopause symptoms	116 (29)	190 (48)	93 (23)
Blockage of your intestines or obstruction	251 (62)	60 (15)	93 (23)
Abnormal cells on your cervix/vagina	308 (75)	78 (19)	25 (6)
Problems with sexual function	114 (29)	186 (46)	101 (25)
Fertility-related concerns	67 (17)	171 (44)	148 (49)

**Fig. 1:**
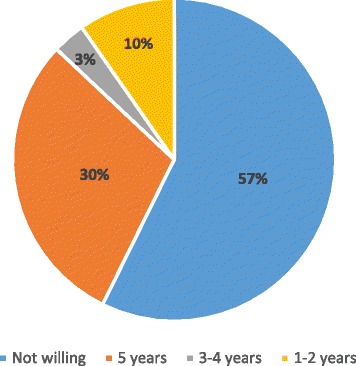
Time after conclusion of treatment at which survivors are willing to transfer care to OB/GYNs. More than half of the participants preferred to have all survivorship care with their oncologists indefinitely

An oncologist was the first choice for a survivorship practitioner in 331 patients (78.6%), followed by internal medicine (10.9%), family practice (3.8%), and OB/GYN (6.7%). Of those patients who identified the oncologist as their first choice, 48.5% reported an OB/GYN being the second choice. More than half of the patients (57.3%) reported being unwilling to transfer surveillance care to an OB/GYN at any time, and 29.7% of patients reported willingness to transfer surveillance care to an OB/GYN after five years (Fig. 1). Only 6.7% of patients felt comfortable transferring care after one year. When evaluated further, women aged greater than 60 years were more likely to report a desire never to leave the oncologist for survivorship follow up (OR 1.53 [95% CI 1.03–2.27], *p* = 0.03). Additionally, a greater proportion of women with ovarian cancer reported being unwilling to leave the oncologist when compared to women with uterine cancer (69.2% vs. 34.2%, *p* < 0.001). There were no differences in preference for survivorship care by insurance type (*p* = 0.11) or distance to physician office (*p* = 0.18). Those women who reported being unwilling to transfer surveillance care to an OB/GYN at any time cited mistrust in the OB/GYN, perceived incorrect or missed diagnoses of cancer by the OB/GYN, greater confidence in the ability of the oncologist to detect a recurrence, and having an established relationship with the oncologist as reasons for their responses.

## Discussion

In this cohort of gynecologic cancer survivors, it is clear that the preference is for oncologists to play an integral part in the survivorship portion of the cancer continuum. There are variable preferences in regards to specific post-treatment medical issues, as well as variation by age and disease type in the willingness to transition care out of the oncologist’s office to either an OB/GYN or other non-cancer specialist. These data suggest that assigning a uniform approach to cancer survivorship may not be feasible for women treated for gynecologic malignancies.

With increasing numbers of cancer survivors expected over the next decade, the need to provide specialized care for this population will become even more pressing. Cancer survivors are a unique group of individuals; not only do they need long-term surveillance for cancer recurrence, but they require management of treatment side effects, rehabilitation, psychosocial counseling, and general health maintenance. As such, coordination of care for these patients may be particularly complicated.

In our cohort, more than 50% of the patients did not feel comfortable going to any other physician but their primary oncologist for survivorship care, and 85% wanted their oncologists to provide screening for recurrent malignancies. This percentage is greater than that reported by medical oncology patients, 61% of whom expected full participation of their oncologists in follow-up for cancer recurrence [3]. Additionally, only 2% of medical oncology patients expected their oncologists to be involved in the treatment of other medical problems [3] – a stark contrast to the gynecologic cancer survivors questioned here, amongst whom 24% preferred their oncologist to take care of their thyroid issues, 19% their hypertension, and 16% their cholesterol. While the medical oncology cohort of patients was much more diverse and included men, the differences in these desires may reflect different experiences of women with gynecologic cancers, and specifically of ovarian cancer patients, who comprised the largest proportion of patients surveyed. It is well-known that the symptoms of ovarian cancer are vague, and there is commonly a delay in diagnosis [10]. Several of the respondents noted on the questionnaire that they did not want to leave the care of the oncologist at any time because there had been a delay in diagnosis of or misdiagnosis prior to the detection of their cancer. They expressed hesitation about returning to a physician who had “missed” their ovarian cancer; concerns about other potential “missed” medical issues may be precluding these patients from embracing transfer to a physician other than the oncologist. Addressing these concerns, and discussing the difficulties in diagnosing ovarian cancer, will be a crucial part of the survivorship process so that patients will ultimately have confidence in continuing care in a non-oncologic setting. Increased collaboration between oncologists and other providers, as well as concerted education efforts regarding signs and symptoms of gynecologic cancers and recurrent disease, would likely be beneficial.

As noted in Table 2, more than 30% of respondents preferred their oncologists to manage thirteen of the 23 (57%) medical topics queried. These issues included not only oncology-specific problems (cancer surveillance, bowel obstruction) but also potentially non-oncology-specific problems (anemia, rectal bleeding). The reliance of patients on their oncologists to manage non-oncologic issues will become problematic in the near future given the growing disparity between the supply of oncologists and the resources required to treat patients with active cancer. In 2007, Erikson et al. [11] conducted a survey of practicing oncologists (including gynecologic oncologists) and fellowship program directors to estimate what the oncology service demand would be by 2020 and the supply of oncologists able to care for these patients. By 2020, the demand was expected to increase by 48%, while the supply of oncologists was expected to increase only 14%. Similar trends have been reported by others [12]. As the numbers of newly diagnosed cancer patients grows, there will be significant limitations on the part of oncologists to address long-term medical issues in survivors and also keep up with the numbers of patients with new diagnoses of cancer. It is incumbent upon oncologists, then, to prepare patients for transition to other types of physicians for long-term survivorship care so that there will be sufficient time to treat patients with active disease.

In our study, the majority of participants had ovarian cancer, which is not the most common gynecologic malignancy. Endometrial cancer, on the other hand, affects nearly twice the number of women that ovarian cancer does [13], yet the participation of women with endometrial cancer was less in this study. All registered survivors with the FWC were eligible to participate, but fewer endometrial cancer patients responded. This calls into question how much women with endometrial cancer identify themselves as cancer survivors or engage in survivorship activities, and does introduce a degree of response bias in this study. Identifying oneself as a survivor is an important concept for endometrial cancer patients to endorse, as interventions designed for cancer survivors, and in particular those focusing on diet and physical activity, would likely be very beneficial for these women [14]. Future efforts to increase involvement of endometrial cancer survivors in survivorship advocacy groups should be encouraged.

Patient age, and specifically age less than 60 years, was significantly associated with willingness to see a benign gynecologist for follow-up. A similar observation has been made in survivors of non-gynecologic malignancies. In a recent study of the preferences for survivorship care and the perceived burden of medical and psychosocial conditions among breast cancer patients, women most interested in participating in a survivorship clinic independent of the primary oncologist were those younger than 57 years of age [15]. Aside from young age, a history of chemotherapy treatment was also associated with a greater desire to participate in a survivorship program. Perhaps patients who do not receive chemotherapy or radiation, by contrast, do not see themselves as having need for a survivorship program. If this is true, it may provide one reason for the lack of endometrial cancer survivor participation in survivor activities, as previously mentioned.

Coordination of care between patient and oncologist, patient and primary care provider, and oncologist and primary care provider are critical to optimize the care of the cancer survivor, and effective and timely communication may ameliorate some of the hesitation on the part of the patient in transitioning away from her oncologist. Cheung et al. [16] reported significant discordance between PCP, oncologist, and patient expectations for survivorship care, with patients anticipating significantly more involvement on the part of the oncologist. However, patient expectations were much more realistic when a discussion about cancer follow-up had occurred prior to transition back to the PCP. An additional finding was that PCPs and oncologists had a high discordance in the perception of their roles in general health maintenance, cancer follow-up, and secondary cancer screening. A follow-up to this study by the same group noted that there was disagreement between PCPs and oncologists regarding the optimal model for delivery of survivorship care [9]. Such a discordance may lead to even poorer communication between oncologist and PCP, as demonstrated by Salz et al. [17]. These authors surveyed PCPs regarding their preferences for information about colorectal cancer survivorship care. Most participants expressed concern about the information they received from treating oncologists about potential long-term effects of chemotherapy (73%) and radiation (67%), screening for other secondary malignancies (78%), and genetic counseling (83%). Increasing communication and establishing relationships between physicians by utilizing survivorship care plans as recommended by the IOM and mandated by the American College of Surgeons Commission on Cancer [2], may help ameliorate some of the patient concerns about transitioning between providers, while simultaneously improving relationships between specialty and generalist physicians [15, 18].

This study is limited by its selection bias. Only patients registered as survivors with the FWC were eligible for participation, and those who responded to the survey voluntarily went to the website to participate. There were no mechanisms by which to ultimately determine a denominator, and thereby a response rate, or if duplicate responses were obtained. Additionally, the instrument used has not previously been validated. However, at the initiation of this study, no validated instrument to assess survivorship preferences for care was available. Presently, there is only one validated instrument in use globally for assessment of survivorship care needs (Supportive Care Needs Survey, SCNS-34) [19]. This tool, however, emphasizes patient-specific needs without assessing patterns of follow-up care. The majority of women who responded to the questionnaire (72%) had private insurance and only a small fraction were uninsured (< 2%), which also introduces selection bias as these are women who had the financial means to participate in survivorship courses and may have different concerns or preferences than women in a lower socioeconomic class. Finally, there was a disproportionate number of younger women than would be expected in the overall gynecologic oncology population, which may be a reflection of either access to the internet or increased capability of technology use. While these limitations reduce the generalizability of our results to a larger population of gynecologic cancer survivors, we believe that as a hypothesis-generating exploratory study, there is much to be learned from this population regarding preferences for post-treatment care. What is clear from our cohort is that gynecologic cancer patients prefer their oncologists to be heavily involved in their care as they enter the survivorship phase of the cancer continuum. However, it is important to note that while the goal of this investigation was to identify preferences, it does not assess whether or not patients would have been dissatisfied if they followed up with their second, third, or even fourth physician choices, or even reluctant to do so. In many cases, due to insurance or geographic reasons, patients may not be able to seek care with their first choice, and thus may be evaluated by a provider the selection of whom is independent of their wishes. Future endeavors in this population should include an evaluation of preferences by race, relationship status, and nativity, as well as special attention to segregation by disease site as our data suggest these patients may be different enough to warrant individual analyses.

As the number of oncologists diminishes in relation to the number of cancer patients, recruiting the assistance of OB/GYNs and PCPs will be imperative to keep up with the needs of the growing numbers of survivors. Early introduction of survivorship programs and resources, and engaging practitioners interested in caring for the special needs of cancer survivors are challenges that oncologists will continue to face. It will also be important to potential consider alternative adjunctive methods to conventional clinic-based survivorship care with physicians, including individualized nursing or advanced practitioner visits or telephone follow-up, both of which have demonstrated acceptability by patients and improved quality of life assessments [20, 21]. Ultimately, by developing a cohesive team approach to the care of these patients and utilizing the skills of various practitioners, successful and effective survivorship care is an attainable goal.

## Conclusion

Our investigation demonstrated that while gynecologic cancer survivors generally prefer that oncology specialists take the lead in their survivorship medical care, there is significant variability in preferences for the specific aspects of that care. Ongoing studies to further evaluate the nuances of gynecologic cancer survivorship are warranted to optimize the care for these patients.

## Appendix

### Patient Preferences for Gynecologic Cancer Survivor Care Survey

1. What is the date today?
  - Free text MM/DD/YYYY
2. Please select the type of cancer you are receiving/have received treatment for:
  - Vulvar
  - Vaginal
  - Cervical
  - Uterine/Endometrial
  - Ovarian/Fallopian Tube/Primary Peritoneal
  - Pregnancy-related cancer/Gestational Trophoblastic Disease
  - Other (with free text)
3. What is your current age?
  - 18–29 years
  - 30–39 years
  - 40–49 years
  - 50–59 years
  - 60–69 years
  - 70–79 years
  - 80 years or older?
4. At what age were you diagnosed with a gynecologic cancer?
  - 19 years or younger
  - 20–29 years
  - 30–39 years
  - 40–49 years
  - 50–59 years
  - 60–69 years
  - 70–79 years
  - 80 years or older
5. Select the region where you currently live:
  - Northeast (Maine, New Hampshire, Vermont, Massachusetts, Rhode Island, Connecticut)
  - Mid-Atlantic (New York, Pennsylvania, New Jersey)
  - South Atlantic (Delaware, Washington DC, Maryland, West Virginia, North Carolina, South Carolina, Georgia, Florida)
  - East South Central (Alabama, Kentucky, Tennessee, Mississippi)
  - West South Central (Louisiana, Arkansas, Oklahoma, Texas)
  - East North Central (Ohio, Michigan, Indiana, Illinois, Wisconsin)
  - West North Central (Minnesota, Iowa, Missouri, Kansas, Nebraska, South Dakota, North Dakota)
  - Mountain (Montana, Wyoming, Colorado, New Mexico, Idaho, Utah, Nevada, Arizona)
  - Pacific (Washington, Oregon, California, Hawaii, Alaska)
  - Other (with free text)
6. Please indicate which of the following insurance plans below best describes your current insurance coverage:
  - Medicaid
  - Medicare
  - Military
  - Private Insurance (PPO, HMO, etc.)
  - Uninsured
  - Other (with free text)
7. Have you previously been treated/are receiving treatment for a cancer recurrence?
  - Yes
  - No
  - Unknown
8. Please indicate if you have received any part of your CANCER care from the following types of doctors:

**Table:**

	**Yes**	**No**	**Unknown**
**Gynecologic Oncologist**			
**Medical Oncologist**			
**Radiation Oncologist**			
**Other Physician**			

9. Who would you consider to be your PRIMARY oncologist?
  - Gynecologic Oncologist
  - Medical Oncologist
  - Radiation Oncologist
  - Other (with free text)

10. Approximately how close to your current residence is the office of the primary oncologist who is treating/has treated your cancer?
  - Less than 20 miles
  - Between 20 and 50 miles
  - Between 50 and 100 miles
  - More than 100 miles
11. Considering distance, ease of getting appointments, childcare costs, and parking/transportation costs, how convenient is it for you to go to (or have gone to) appointments at your primary oncologist’s office?
  - Very inconvenient
  - Slightly inconvenient
  - Neither convenient or inconvenient
  - Slightly convenient
  - Very convenient

12. Which of the following statements is MOST true for you?
  - My primary oncologist’s office is closer to my home that my general gynecologist’s office.
  - My general gynecologist’s office is closer to my home than my primary oncologist’s office.
  - The distance to my primary oncologist’s office and my gynecologist’s office is about the same.
  - I do not have a general gynecologist.

13. Do you currently seek follow-up care for your cancer from someone other than your primary oncologist or general gynecologist?
  - No
  - Yes (If yes, with whom?)

14. If you were able to see any doctor for follow-up care, including cancer surveillance and general health conditions, regardless of financial limitations, which kind of doctor would that be? Please indicate your 1st choice, 2nd choice, 3rd choice, and 4th choice. Select only one answer per physician category.

**Table:**

	1st Choice	2nd Choice	3rd Choice	4th Choice
Oncologist (any type)				
General internal medicine specialist				
General gynecologist				
Family Practitioner				
Other				

15. For each of the following medical problems, please select if you would prefer for your general gynecologist or oncologist to treat you for that problem. If you have no preference, please select “no preference”.

**Table:**

	Gynecologist	Oncologist	No Preference
Diabetes			
Thyroid Problems			
Quitting Smoking			
Weight Loss			
High Cholesterol			
Depression or Anxiety			
High Blood Pressure			
Anemia			
Kidney Problems			
Problems Urinating			
Problems with Bowel Movements			
Passing stool from your vagina			
Passing urine from your vagina			
Bleeding from your rectum			
Blood in your urine			
Cancer recurrence			
Low bone density			
Excessive swelling in your legs (lymphedema)			
Menopause symptoms			
Blockage of your intestines/obstruction			
Abnormal cells on your cervix or vagina			
Problems with sexual function			
Fertility-related concerns			

16. How many years after completing treatment for your cancer would you be willing to see a GENERAL GYNECOLOGIST instead of your oncologist for follow-up cancer surveillance and general medical care?
  - 1 year
  - 2 years
  - 3 years
  - 4 years
  - 5 years or more
  - Not willing (if not willing, provide an explanation why)

## Abbreviations

FWC: Foundation for Women’s Cancer
HMO: Health Maintenance Organization
IOM: Institute of Medicine
OB/GYN: Obstetrician/Gynecologist
PCP: Primary Care Provider
PPO: Preferred Provider Organization
SCNS-34: Supportive Care Needs Survey-34
